# Serosurvey of selected reproductive pathogens in domestic ruminants from Upper Egypt

**DOI:** 10.3389/fvets.2023.1267640

**Published:** 2023-10-23

**Authors:** Shimaa Ismail Farag, David Cano-Terriza, Moisés Gonzálvez, Doaa Salman, Nasr-Eldin M. Aref, Murad A. Mubaraki, Débora Jiménez-Martín, Ignacio García-Bocanegra, Ehab Kotb Elmahallawy

**Affiliations:** ^1^Department of Animal Medicine, Faculty of Veterinary Medicine, Sohag University, Sohag, Egypt; ^2^Departamento de Sanidad Animal, Grupo de Investigación en Sanidad Animal y Zoonosis (GISAZ), UIC Zoonosis y Enfermedades Emergentes ENZOEM, Universidad de Córdoba, Córdoba, Spain; ^3^CIBERINFEC, ISCIII CIBER de Enfermedades Infecciosas, Instituto de Salud Carlos III, Madrid, Spain; ^4^Departamento de Sanidad Animal, Facultad de Veterinaria, Campus de Excelencia Internacional Regional “Campus Mare Nostrum”, Universidad de Murcia, Murcia, Spain; ^5^Department of Animal Medicine, Faculty of Veterinary Medicine, Assiut University, Assiut, Egypt; ^6^Clinical Laboratory Sciences Department, College of Applied Medical Sciences, King Saud University, Riyadh, Saudi Arabia; ^7^Department of Zoonoses, Faculty of Veterinary Medicine, Sohag University, Sohag, Egypt

**Keywords:** seroprevalence, *Toxoplasma gondii*, *Neospora caninum*, *Coxiella burnetii*, ruminants, Egypt

## Abstract

Toxoplasmosis, neosporosis, and Q fever are among the most important abortifacient diseases in ruminants worldwide. These diseases result in huge economic losses in livestock besides the fact that some of are of public health concern. The present study aimed to update the data about the current seroepidemiological situation of these diseases in Upper Egypt. A total of 411 blood samples were collected from small and large ruminants and serologically tested against the presence of *T. gondii*, *N. caninum*, and *C. burnetii*. Generalized estimating equation (GEE) models were performed to assess the potential risk factors associated with the exposure to these pathogens. The overall seroprevalence of *T. gondii* was 47.9% (197/411) with an individual seropositivity of 59.4% (63/106), 58.6% (17/29), 38.8% (54/139) and 46% (63/137) in cattle, buffalo, sheep and goats, respectively. Meanwhile, 9.7% (38/411) of the examined animals were tested positive for anti-*N. caninum* antibodies, with an individual seropositivity of 13.2% (12/106), 34.5% (10/29), 8.6% (12/139) and 2.9% (4/137) in cattle, buffalo, sheep and goats, respectively. Furthermore, the overall prevalence of antibodies against *C. burnetii* was 17.3% (63/411), and exposure to this pathogen was detected in 4.7% (5/106) of cattle, 19.3% (20/129) of sheep, 29.2% (38/130) of goats but none of the examined buffalo were found to be seropositive. A total of 12.1% (50/411) of the examined animals showed co-exposure to at least two of the tested pathogens. Regarding the potential risk factors, there were statistically significant differences among species in the frequency of exposure to the three tested pathogens. Age (> 6 months) was also shown to be a significant risk factor associated with *T. gondii* exposure. The results obtained provided updated information about the occurrence of three of the main reproductive pathogens in Upper Egypt. The high seropositivity values found for the tested zoonotic pathogens in most of the analyzed ruminant species suggest the necessity of performing additional in-depth studies to evaluate the epidemiology of these pathogens in the study area.

## 1. Introduction

Toxoplasmosis, neosporosis and Q fever are important abortifacient diseases associated with serious reproductive disorders in domestic ruminants and severe economic losses in livestock worldwide (1). They are caused by *Toxoplasma gondii, Neospora caninum*, (intracellular protozoan belongs phylum Apicomplexa) and *Coxiella burnetii* (obligate intracellular bacterium; family Coxiellaceae), respectively (2). *Toxoplasma gondii* and *N. caninum* have similar indirect life cycles, including a wide range of warm-blooded vertebrates as intermediate hosts and Felidae (*T. gondii*) or Canidae (*N. caninum*) as definitive hosts. These parasites are mainly transmitted through the ingestion of food and water contaminated with sporulated oocysts or congenitally (3–5). Although other infection routes have been reported for *C. burnetti* (e.g., vector and aerosol-borne transmission), contaminated food or water also play a key role in the epidemiology of this bacterium (6).

Nowadays, *T. gondii* is considered a major cause of abortion, stillbirth and weak lambs in sheep and cattle (7, 8), while *N*. *caninum* is the main cause of abortion and/or neonatal mortality in cattle worldwide (9). The hallmark of *C. burnetii* in domestic ruminants is late-term abortion, with rates as high as 80–90% (10, 11). In addition, other reproductive disorders of *C. burnetii* in cattle, goat and sheep include small, weak offspring, retained placenta and chronic metritis (8, 9, 12).

Even though most of the *T. gondii* and up to 60% of the *C. burnetti* infections are usually asymptomatic in humans (5, 13, 14), these two pathogens are of public health concern. Toxoplasmosis can lead to abortion, important neuromuscular diseases in immunocompromised people and even death (5). In addition, Q fever may be presented as acute febrile self-limited disease with headache, myalgia, pneumonia or hepatitis (15). Meanwhile, despite the considerable veterinary and economic importance of *N*. *caninum*, currently it is not considered to be relevant for human health (16).

Egypt, considered a developing country, has an estimated population of 16.3 million ruminants, which represents an important driver for the economy of rural areas (17). The traditional husbandry in Egypt is based on small holders who might own different animal species together, including cattle, buffalo and/or small ruminants, donkeys and camels usually reared nearby the other species too. Additionally, these smallholders commonly have one or more watchdogs in the herds, and stray or domestic cats usually roam freely around the farms. As a result, and taking into consideration the low socioeconomic conditions of the majority of Egyptian villages, hygienic and sanitary conditions are often inadequate in most of the farms.

Providing periodical update about the occurrence of the transmissible diseases is critical for implementation of effective control measures against infection. There are some reports describing the occurrence of *T. gondii*, *N. caninum* and *C. burnetti* in Northern part of Egypt (Lower Egypt) which are listed in Table 1 (18–48). However, there is a lack of information about the current epidemiological scenario at the Southern part of the country (Upper Egypt), particularly in Sohag governorate, which has obvious importance for livestock production besides its agricultural nature (49). Therefore, the aim of this study was to assess the seroprevalence and risk factors associated with domestic ruminants’ exposure to these reproductive pathogens in Sohag governorate, Egypt.

**Table 1:** Seroprevalence of studied pathogens in domestic ruminants in Egypt.

Pathogen	Host	Governorate	Area	Detection method	Frequency % (no. pos./total)	Reference
*Toxoplasma gondii*	Cattle	Assuit	Upper Egypt	LAT, ELISA	32.1 (18/56) / 73.2 (41/56)	(18)
	Cattle	Qena/ Sohag	Upper Egypt	LAT, ELISA	29.2/ 28.2	(19)
	Cattle	Qena/ Cairo/ Sohag/ Dakahlia	Upper & Lower Egypt	ELISA (milk)	2.4 (3/126)	(20)
	Cattle	Sharkia	Lower Egypt	ELISA	10.8 (10/93)	(21)
	Cattle	Alexandria and Matrouh	Lower Egypt	ELISA	13.5 (14/104)	(22)
	Cattle	Beheira	Lower Egypt	ELISA	5.3 (19/358)	(23)
	Cattle	Menoufia	Lower Egypt	ELISA	3.1 (8/262)	(24)
	Buffalo	Assuit	Upper Egypt	LAT, ELISA	74.5(41/55) / 20 (11/55)	(18)
	Buffalo	Giza	Lower Egypt	MAT	22.6 (36/160)	(25)
	Buffalo	Gharbia	Lower Egypt	MAT	16.0 (12/75)	(26)
	Buffalo	Cairo, Giza & Kalubiya	Lower Egypt	ELISA	17.1 (7/41)	(27)
	Buffalo	Dakahlia	Lower Egypt	ELISA (milk)	0.0 (0/16)	(20)
	Buffalo	Menoufia	Lower Egypt	ELISA	8.2 (20/244)	(24)
	Sheep	Assuit	Upper Egypt	LAT (raw milk)	39.7 (23/58)	(28)
	Sheep	Assuit	Upper Egypt	LAT, ELISA	44.0 (22/50) / 86 (43/50)	(18)
	Sheep	Luxor	Upper Egypt	ELISA	40.2 (37/92)	(29)
	Sheep	Quena, Kafr El Sheikh and Minoufiya	Upper & Lower Egypt	LAT, ELISA	47.8 (53/111) / 51.4 (57/111)	(19)
	Sheep	Dakahlia, Beni Suef, Qena, Red Sea	Upper & Lower Egypt	ELISA	35.6 (85/239)	(20)
	Sheep	Dakahlia	Lower Egypt	ELISA (milk)	66.7 (12/18)	(20)
	Sheep	Alexandria and Matrouh	Lower Egypt	ELISA	43.8 (63/144)	(22)
	Sheep	Dakahlia	Lower Egypt	LAT, IHAT, ELISA	41.7 (122/292) / 66.1(173/292) / 62 (181/292)	(30)
	Sheep	Alfayium	Lower Egypt	ELISA, Dye test	98.4 (61/62)	(31)
	Sheep	Cairo, Giza and Al-Sharkia	Lower Egypt	ELISA/OnSite Toxo IgG/IgM Rapid test cassettes	51.3 (58/113) / 58.4 (66/113)	(32)
	Sheep	Cairo, Giza, Dakahlia and Sharkia	Lower Egypt	IFA, ELISA	4.1 (16/398) / 26 (103/398)	(33)
	Sheep	Giza	Lower Egypt	IHAT	47.5 (152/320)	(34)
	Goat	Assuit	Upper Egypt	LAT (raw milk)	38.3 (18/47)	(28)
	Goat	Assuit	Upper Egypt	LAT, ELISA	47.4 (27/57) / 87.7 (50/57)	(18)
	Goat	Luxor	Upper Egypt	ELISA	34.8 (32/92)	(29)
	Goat	Quena, Kafr El Sheikh and Minoufiya	Upper & Lower Egypt	LAT, ELISA	35.1 (33/94) /39.4 (37/94)	(19)
	Goat	Dakahlia, Beni Suef, Qena, Red Sea	Upper & Lower Egypt	ELISA	66.9 (81/121)	(20)
	Goat	Dakahlia	Lower Egypt	ELISA (milk)	81.8 (9/11)	(20)
	Goat	Alexandria and Matrouh	Lower Egypt	ELISA	27.9 (31/111)	(22)
	Goat	Dakahlia	Lower Egypt	LAT, IHAT, ELISA	49.4 (40/81) / 64.2 (52/81) / 50.6 (41/81)	(30)
	Goat	Lfayium	Lower Egypt	ELISA, Dye test	41.7 (10/24)	(31)
	Goat	Cairo, Giza and Al-Sharkia	Lower Egypt	ELISA/OnSite Toxo IgG/IgM Rapid test cassettes	41.0 (39/95) / 45.2 (43/95)	(32)
	Goat	Dakahlia	Lower Egypt	IFA, ELISA	62.0 (62/100)	(33)
	Goat	Giza	Lower Egypt	MAT	44.3 (102/230)	(25)
	Goat	Kalubyia	Lower Egypt	IHAT, MAT	35.4 (17/48) / 22.9 (11/48)	(35)
	Goat	Sharkia	Lower Egypt	IHAT	16.0 (8/50)	(36)
*Neospora caninum*	Cattle	Qena/ Sohag	Upper Egypt	ELISA	18.9 (57/301)	(37)
	Cattle	Al-Sharkia	Lower Egypt	ELISA	20.4 (19/93)	(21)
	Cattle	Dakahlia	Lower Egypt	ELISA (milk)	26.2**(**33/126)	(20)
	Cattle	Beheira	Lower Egypt	ELISA	24.6 (88/358)	(23)
	Cattle	Menoufia	Lower Egypt	ELISA	14.9 (39/262)	(24)
	Cattle	Kafrelsheikh	Lower Egypt	ELISA	38.0 (35/92)	(38)
	Buffalo	Cairo	Lower Egypt	Neospora agglutination test	68.0 (51/75)	(26)
	Buffalo	Menoufia	Lower Egypt	ELISA	13.5 (33/244)	(24)
	Sheep	Luxor	Upper Egypt	ELISA	6.5 (6/92)	(29)
	Sheep	Alexandria, Gharbia, Menofia, Kalubiya	Lower Egypt	ELISA	8.6 (37/430)	(39)
	Sheep	Nile Delta regions	Lower Egypt	Direct agglutination test	36.1 (73/202)	(40)
	Sheep	Dakahlia, Beni Suef, Qena, Red Sea	Upper & Lower Egypt	ELISA	15.5 (37/239)	(20)
	Goat	Dakahlia, Beni Suef, Qena, Red Sea	Upper & Lower Egypt	ELISA	5.0 (6/121)	(20)
	Goat	Nile Delta regions	Lower Egypt	Direct agglutination test	35.2 (31/88)	(40)
*Coxiella burnetii*	Cattle	Assuit	Upper Egypt	IFAT, ELISA	45.3 (34/75) / 50.7 (38/75)	(41)
	Cattle	25 governorates	Upper & Lower Egypt	ELISA	19.3 (162/840)	(42)
	Cattle	Dakahlia, Damietta and Port Said.	Lower Egypt	ELISA	13.2 (158/1194)	(43)
	Cattle	Nile Delta	Lower Egypt	ELISA	19.8 (95/480)	(44)
	Cattle	Giza, Cairo and Fayoum	Lower Egypt	ELISA	13.0 (7/54)	(45)
	Buffalo	25 governorates	Upper & Lower Egypt	ELISA	11.2 (34/304)	(42)
	Sheep	Assuit	Upper Egypt	IFAT, ELISA	56.0 (28/50) / 60.0 (30/50)	(41)
	Sheep	El Minya	Upper Egypt	ELISA	25.7 (28 of 109)	(46)
	Sheep	25 governorates	Upper & Lower Egypt	ELISA	8.9 (64/716)	(42)
	Sheep	8 governorates	Upper & Lower Egypt	ELISA	22.5 (95/420)	(47)
	Sheep	Kalubiya	Lower Egypt	IFAT	23.0 (23/100)	(48)
	Sheep	Giza, Cairo and Fayoum	Lower Egypt	ELISA	32.7 (18/55)	(45)
	Goat	Assuit	Upper Egypt	IFAT, ELISA	45.7 (16/35) / 51.4 (18/35)	(41)
	Goat	El Minya	Upper Egypt	ELISA	28.2 (11/39)	(46)
	Goat	25 governorates	Upper & Lower Egypt	ELISA	6.8 (21/311)	(42)
	Goat	8 governorates	Upper & Lower Egypt	ELISA	23.1 (74/320)	(47)
	Goat	Kalubiya	Lower Egypt	IFAT	27.0 (27/100)	(48)
	Goat	Giza, Cairo and Fayoum	Lower Egypt	ELISA	23.3 (7/30)	(45)

## 2. Materials and methods

### 2.1. Study design and sample collection

A cross-sectional study was carried out in domestic ruminant species in Sohag governorate (Upper Egypt) (26.56°N 31.7°E) between May and September 2021. The climate in the study area is characterized as desertic, with no rainfall during the year except little in winter, and a relative humidity ranging from 60% to less than 30% (50).

A total of 411 animals from small stakeholders were sampled, including 106 cattle, 29 buffalos, 139 sheep, and 137 goats in 13 different municipalities. The sample size was calculated using WinEpiscope 2.0.^1^ In consideration of the number of domestic ruminants in the study area (n > 10,000), an estimated prevalence of 50%, which provides the highest simple size in studies with unknown prevalence (51), the desired absolute precision was set at ±5% and confidence level at 95%, resulting in 385 animals to be sampled and a total of 411 animals from small stakeholders were finally included in the study. Blood samples were collected by jugular vein puncture using sterile tubes without anticoagulant (Vacutainer®, Becton-Dickinson, USA). Samples were transported to the laboratory (Department of Animal Medicine, Sohag University, Egypt) under refrigerated conditions (4–6°C) within 24–48 h following collection, then centrifugated at 400 *g* for 15 min to obtain serum, and preserved at −20°C until analysis. Information about each animal, including species, sex, age and some other general clinical information such a pregnancy, presence of ectoparasites, history of abortion, diarrohea and fever, were collected whenever possible (Table 2). None of surveyed animals was vaccinated against toxoplasmosis, neosporosis or Q fever.

**Table 2:** Distribution of variables associated with seropositivity of studied pathogens in ruminants in Sohag governorate.

		*Toxoplasma gondii*			*Neospora caninum*			*Coxiella burnetti*		
Variable	Categories	Positives/overall	%	*p value*	Positives/overall	%	*p value*	Positives/overall	%	*p value*
Species	Buffalo	17/29	58.6	0.008	10/29	34.5	<0.001	0/27	0.0	<0.001
	Cattle	63/106	59.4		14/106	13.2		5/106	4.7	
	Goat	63/137	46.0		4/137	2.9		38/130	29.2	
	Sheep	54/139	38.8		12/139	8.6		25/129	19.4	
Age	< 6 months	23/67	34.3	0.010	4/67	6.0	0.183	13/64	20.3	0.300
	> 6 months	174/344	50.6		36/344	10.5		55/328	16.8	
Gender	Female	148/287	51.6	0.016	29/287	10.1	0.426	50/276	18.1	0.322
	Male	49/124	39.5		11/124	8.9		18/116	15.5	
Pregnancy	No	116/232	50.0	0.173	26/232	11.2	0.152	38/223	17.0	0.222
	Yes	32/55	58.2		3/55	5.5		12/53	22.6	
Ectoparasites	No	180/370	48.6	0.24	32/370	8.6	0.033	63/352	17.9	0.271
	Yes	17/41	41.5		8/41	19.5		5/40	12.5	
Abortion	No	145/281	51.6	0.628	27/281	9.6	0.115	49/270	18.1	0.702
	Yes	3/6	50.0		2/6	33.3		1/6	16.7	
Fever	No	189/385	30.8	0.053	3/26	11.5	0.475	6/25	24.0	0.253
	Yes	8/26	49.1		37/385	9.6		62/367	16.9	
Diarrhea	No	185/386	47.9	0.578	36/386	9.3	0.217	61/367	16.6	0.121
	Yes	12/25	48.0		4/25	16.0		7/25	28.0	
Anorexia	No	116/283	41.0	<0.001	28/283	9.9	0.513	48/268	17.9	0.390
	Yes	81/128	63.3		12/128	9.4		20/124	16.1	
Cachexia	No	130/306	42.5	<0.001	31/306	10.1	0.401	57/293	19.5	0.037
	Yes	67/105	63.8		9/105	8.6		11/99	11.1	

### 2.2. Serological analysis

The presence of *T. gondii* antibodies was detected using the modified agglutination test (MAT) as previously described (Dubey and Desmonts, 1987). Sera with titers ≥1:25 were considered positive. This technique has been employed broadly for the diagnosis of antibodies against *T. gondii* in both domestic and wildlife ruminants (Dubey, 2022). Sera were also analyzed to detect the presence of antibodies against *N. caninum* and *C. burnetii* using two commercial ELISA kits (ID Screen® *Neospora caninum* Competition and ID Screen® Q fever Indirect Multi-species, France) according to the manufacturer’s recommendation. The sensitivity and specificity values provided by the manufacturer for both ELISA were 100%. These ELISA kits have been used previously in different studies of domestic and wild ruminant species (52–55).

### 2.3. Statistical analysis

The individual seroprevalence against toxoplasmosis, neosporosis and Q fever was calculated from the ratio of seropositive samples to the total number of animals examined with a 95%CI. Associations between explanatory variables and serological results to the three pathogens analyzed (dependent variables) were performed using the Pearson’s chi-square or Fisher’s test, as required. Then, explanatory variables with *value of p* < 0.10 were selected for multivariate analysis. Collinearity between variables was also calculated using the Cramer’s V coefficients. Finally, generalized estimating equation (GEE) models were carried out for each tested pathogen. The number of seropositive animals was assumed to follow a binomial distribution and “municipality” was included as a random effect. Values with *p* < 0.05 were considered statistically significant. SPSS 25.0 software (IBM Corp., Armonk, NY, United States) was used to perform statistical analyses.

## 3. Results

Table 2 depicts the results of serosurvey for the studied pathogens besides pointing out some other general clinical information. As shown, *T. gondii* had overall seroprevalence of 47.9% (197/411; 95%CI: 43.1–52.8%). *T. gondii* antibodies against were detected in 59.4% (63/106) of cattle, 58.6% (17/29) buffaloes, 46.0% (63/137) goats and 38.8% (54/139) sheep. In addition, 9.7% (40/411; 95%CI: 6.9–12.6%) of the ruminants sampled showed anti-*N. caninum* antibodies. The highest seroprevalence was observed in buffaloes (34.5%; 10/29), followed by cattle (13.2%; 14/106), sheep (8.6%; 12/139) and goats (2.9%; 4/137). Finally, seropositivity of *C. burnetii* was detected in 17.3% (68/392; 95%CI: 13.6–21.1%) of the sampled ruminants, with a seropositivity of 29.2% (38/130) in goats, 19.4% (25/129) in sheep and 4.7% (5/106) in cattle. Anti-*C. burnetii* antibodies were no found in buffaloes (0/29). In relation to the co-exposure cases (Table 3), 15.3% (63/411) of the examined animals were found to be co-exposed to at least two of the tested pathogen; 29 (7.1%) animals had antibodies against both *T. gondii* and *C. burnetii,* 24 (5.8%) showed positive result to both *T. gondii* and *N. caninum* antibodies, 6 (1.5%) had antibodies against *N. caninum and C. burnetii*, and four (1.0%) individuals were found co-exposed by the three tested pathogens.

**Table 3:** Co-exposure of surveyed ruminants’ species with selected reproductive pathogens in Sohag governorate (Upper Egypt).

Pathogen	Cattle (*n* = 106)	Buffalo (*n* = 29)	Sheep (*n* = 139)	Goat (*n* = 137)	Total (%)
	No. positive (%)	No. positive (%)	No. positive (%)	No. positive (%)	No. positive (%)
*T. gondii* + N. *caninum*	11 (10.3)	5 (17.2)	6 (4.3)	2 (1.5)	24 (5.8)
*N. caninum + C. burnetii*	1 (0.9)	0 (0%)	4 (2.9)	1 (0.7)	6 (1.5)
*T. gondii + C. burnetii*	3 (2.8)	0 (0%)	8 (5.8)	18 (13.1)	29 (7.1)
*T. gondii + N. caninum + C. burnetii*	1 (0.9)	0 (0%)	2 (1.4)	1 (0.7)	4 (0.9)
Total	16 (15.1)	5 (17.2)	20 (14.4)	22 (16.1)	63 (15.3)

The independent variables selected in the bivariate analysis are summarized in Tables 2, 4. A total of six, two and two explanatory variables were selected (*p* < 0.10) for the multivariate analysis of *T. gondii*, *N. caninum* and *C. burnetti*, respectively. The final GEE model revealed two potential factors associated with *T. gondii* infection in ruminants: species (buffalo and cattle) and age (> 6 months). In addition, the multivariate analysis showed that species was also a risk factor related to *N. caninum* (buffalo, cattle and sheep) and *C. burnetti* (sheep and goat) exposure (Table 4).

**Table 4:** Data of the generalized estimating equation (GEE) model of the potential risk factors associated with pathogens exposure in domestic ruminants in Sohag governorate (Upper Egypt).

Pathogen	Variable	Categories	*B*	*p value*	OR	95% CI
*Toxoplasma gondii*	Species	Buffalo	0.96	0.01	2.60	1.30–5.20
		Cattle	0.97	<0.001	2.63	1.82–3.80
		Goat	0.38	0.08	1.46	0.96–2.22
		Sheep	*	*	*	*
	Age	> 6 months	0.85	0.009	2.36	1.24–4.45
		< 6 months	*	*	*	*
*Neospora caninum*	Species	Buffalo	2.88	<0.001	17.81	9.16–34.65
		Cattle	1.63	<0.001	5.11	2.52–10.37
		Sheep	1.16	0.001	3.19	1.62–6.29
		Goat	*	*	*	*
*Coxiella burnetii*	Species	Sheep	1.47	<0.001	4.36	1.76–10.81
		Goat	1.89	0.001	6.64	1.99–22.11
		Cattle	*	*	*	*

## 4. Discussion

The present work revealed important baseline information about the seroprevalence of *T. gondii*, *N. caninum* and *C. burnetti* in Sohag governorate, Upper Egypt. Moreover, this work provides novel information about potential the co-exposure between these pathogens in the Upper part of the country. The study also provides updated information about the circulation of the three reproductive pathogens in domestic ruminants throughout the country (Table 1).

In relation to *T. gondii*, the high individual seroprevalence values obtained in cattle (59.4%), buffalo (58.6%), sheep (38.8%) and goats (46%) in our study indicate this parasite is widespread in Upper Egypt, which can be of animal and public health concern. The seroprevalence values falls within the previously reported range for this protozoa (20.0–87.7%) in small and large ruminants from Upper Egypt (Table 1). However, the seroprevalence of *T. gondii* in sheep in our study is slightly lower than those previously found in the study area (39.7–86%) (Table 1). Lower seroprevalence rate of *T. gondii* have also been previously reported in both small and large ruminants in Lower Egypt (0–22.6%) (Table 1). Moreover, the seroprevalence of *T. gondii* in goats falls within the reported range (16–81.8%) in this species in several governorates in Lower Egypt (Table 1).

We identified two risk factors (species and age) associated with *T. gondii* exposure. Seropositivity to *T. gondii* was significantly higher in large ruminants (buffalo and cattle) compared to small ruminants (goats and sheep). This may be attributed to the fact that cattle and buffaloes in this province are usually reared indoor and the tendency of the Egyptian owners to have cats at their homes which may have access to the animals or their feed (12). The risk factor analyses also showed the age as a risk factor associated with *T. gondii* exposure (Table 4). In this sense, the seroprevalence of *T. gondii* was higher in ruminants older than 6 months (50.6%) compared to young ones (34.3%). This finding is consistent with previous investigations (22, 56–59) reporting that a higher seroprevalence of *T. gondii* among older ages indicates that the contact with the pathogen and the persistence of antibodies increases with age (60, 61).

To author’s knowledge, epidemiological studies assessing *N. caninum* in Upper Egypt are limited. The seroprevalence rate obtained in the present work ranged between 2.9% in goat and 34.5% in buffalo (Table 3). The high differences between species, which was shown to be a risk factor for *N. caninum* exposure, are in line with those reported in the scientific literature not only in Egypt (Table 1) but also worldwide (8.6–68.0%) (9, 62). However, our study revealed the lowest seroprevalence rates of *N. caninum* obtained in small ruminant species in Upper and Lower Egypt so far (Table 1). This finding might be due to the variation in management and feeding practices of small ruminants which is usually based on pasture grazing besides the presence of a lot of stray dogs which in turn can contaminate feed and water and results in higher exposure to infection (9, 62).

Concerning *C. burnetii*, high difference between ruminant species were also found in the seropositivity to this zoonotic bacterium, being significantly higher in small ruminant species (29.2 and 19.3% in goat and sheep, respectively) than in large ruminants (0.0 and 4.7% in buffalo and cattle, respectively). Similarly, previous studies carried out in Upper Egypt revealed that the prevalence of antibodies against this pathogen was higher in small ruminants (25.7–60%) compared to cattle and buffalo (11.2–19.3%) (Table 1). In contrast, our survey reported lower seroprevalence values of *C. burnetti* in small and large ruminants than those reported in other studies in Upper and Lower Egypt (6.8 to 27%), respectively (Table 1). Regarding the risk factors analysis, the seropositivity of *C. burnetii* in small ruminants was significantly higher than in large ruminants, which came in stark contrast with some previous reports from Egypt (63). This finding could be explained by the nature of grazing of small ruminants or by differences in the systems of management in this area, where large ruminants are mostly kept indoor, and therefore small ruminants could be more exposed to this pathogen along their life (64).

Interestingly, the present study reports multiple cases of co-exposure by *T. gondii, N. caninum* and/or *C. burnetti*. To the best of our knowledge, this is the first seroepidemiological study evaluating jointly co-exposure of the three tested reproductive pathogens in different domestic ruminant species in Egypt. Metwally et al. (23) detected 1.9% (7/358) of *T. gondii* and *N. caninum* co-exposure in cattle in Beheira governorate in Lower Egypt. Similarly, Aboelwafa et al. (29) reported a co-exposure rate of 4.3% (4/92) of *T. gondii* and *N. caninum* in sheep in Luxor, Upper Egypt. Co-infections with *T. gondii, N. caninum* and *C. burnetii* is usually resulting in lower immunity, increased the risk of abortions, fetal losses and abnormalities which consequently leads to huge economic losses (65). Additional studies are warranted to assess the implications of co-infections by reproductive pathogens in livestock in the study region.

## 5. Conclusion

The present study provides updated seroepidemiological information about the circulation of *T. gondii*, *N. caninum* and *C. burnetti* in four domestic ruminant species to Upper Egypt. To author’s knowledge, the present work is considered the first seroepidemiological study documented the co-exposure of the three tested reproductive pathogens in different domestic ruminant species in Egypt. The circulation of the different selected pathogens was not homogeneous among the analyzed ruminant populations. The seroprevalence values of the tested zoonotic pathogens indicate a relevant epidemiological role of domestic ruminants in the maintenance of these pathogens. The present study point out the importance of improvement of the surveillance programs monitoring the circulation of reproductive pathogens at the domestic-human interface and the role of application of strict hygienic and biosecurity measures to control the infection in Upper Egypt. These measures should include control of access of dogs and cats to the farms, to ruminants rearing areas combined with application of proper vaccination programs to reduce the transmission of these pathogens at this area. Additional molecular and epidemiological surveys addressing the circulation of these reproductive pathogens at a large scale are needed to investigate both their economic and productive impact as well as the sanitary implications for animal and human health in Egypt. Further studies are also suggested to detect the mentioned pathogens on milk samples, meat juice with blood samples for explore the potential zoonotic link and the potential genetic relatedness of circulating strains.

## Data availability statement

The original contributions presented in the study are included in the article/supplementary material, further inquiries can be directed to the corresponding authors.

## Ethics statement

Ethical approval was not required for the study involving animals in accordance with the local legislation and institutional requirements because The collection of blood samples analysed in the present study was part of the official Animal Health Campaigns. Therefore, no ethical approval was necessary.

## Author contributions

SF: Conceptualization, Data curation, Formal analysis, Investigation, Methodology, Validation, Visualization, Writing – original draft, Writing – review & editing. DC-T: Conceptualization, Data curation, Formal analysis, Investigation, Methodology, Software, Supervision, Validation, Visualization, Writing – original draft, Writing – review & editing. MG: Data curation, Formal analysis, Methodology, Software, Supervision, Validation, Visualization, Writing – original draft, Writing – review & editing. DS: Conceptualization, Data curation, Project administration, Supervision, Validation, Visualization, Writing – original draft. N-EA: Conceptualization, Supervision, Validation, Visualization, Writing – original draft. MM: Data curation, Formal analysis, Funding acquisition, Resources, Software, Writing – original draft. DJ-M: Data curation, Formal analysis, Investigation, Methodology, Software, Validation, Writing – original draft, Writing – review & editing. IG-B: Conceptualization, Data curation, Formal analysis, Funding acquisition, Investigation, Project administration, Resources, Supervision, Validation, Visualization, Writing – original draft, Writing – review & editing. EE: Conceptualization, Data curation, Formal analysis, Funding acquisition, Resources, Validation, Writing – original draft, Writing – review & editing.

## Footnotes

^1^http://www.clive.ed.ac.uk/winepiscope/
